# An Exploratory Meta-Analytic Review on the Empirical Evidence of Differential Learning as an Enhanced Motor Learning Method

**DOI:** 10.3389/fpsyg.2021.533033

**Published:** 2021-05-07

**Authors:** Bruno Tassignon, Jo Verschueren, Jean-Pierre Baeyens, Anne Benjaminse, Alli Gokeler, Ben Serrien, Ron Clijsen

**Affiliations:** ^1^Human Physiology and Sports Physiotherapy Research Group, Faculty of Physical Education and Physiotherapy, Vrije Universiteit Brussel, Brussels, Belgium; ^2^Experimental Anatomy Research Group, Faculty of Physical Education and Physiotherapy, Vrije Universiteit Brussel, Brussels, Belgium; ^3^Department of Physiotherapy, International University of Applied Sciences THIM, Landquart, Switzerland; ^4^Faculty of Applied Engineering, Universiteit Antwerpen, Antwerp, Belgium; ^5^Center for Human Movement Sciences, University Medical Center Groningen, University of Groningen, Groningen, Netherlands; ^6^School of Sport Studies, Hanze University Groningen, Groningen, Netherlands; ^7^Exercise Science and Neuroscience Unit, Department Exercise and Health, Faculty of Science, University of Paderborn, Paderborn, Germany; ^8^Amsterdam Collaboration on Health and Safety in Sports, Amsterdam Universitair Medische Centra, Department of Public and Occupational Health, Amsterdam Movement Sciences, Vrije Universiteit Amsterdam, Amsterdam, Netherlands; ^9^Epidemiology and Public Health, Sciensano, Brussels, Belgium; ^10^Rehabilitation Research Laboratory 2rLab, Department of Business Economics, Health and Social Care, University of Applied Sciences and Arts of Southern Switzerland, Landquart/Manno, Switzerland

**Keywords:** meta-analysis, contextual interference, sports, variability, motor learning, differential learning

## Abstract

**Background:** Differential learning (DL) is a motor learning method characterized by high amounts of variability during practice and is claimed to provide the learner with a higher learning rate than other methods. However, some controversy surrounds DL theory, and to date, no overview exists that compares the effects of DL to other motor learning methods.

**Objective:** To evaluate the effectiveness of DL in comparison to other motor learning methods in the acquisition and retention phase.

**Design:** Systematic review and exploratory meta-analysis.

**Methods:** PubMed (MEDLINE), Web of Science, and Google Scholar were searched until February 3, 2020. To be included, (1) studies had to be experiments where the DL group was compared to a control group engaged in a different motor learning method (lack of practice was not eligible), (2) studies had to describe the effects on one or more measures of performance in a skill or movement task, and (3) the study report had to be published as a full paper in a journal or as a book chapter.

**Results:** Twenty-seven studies encompassing 31 experiments were included. Overall heterogeneity for the acquisition phase (post-pre; *I*^2^ = 77%) as well as for the retention phase (retention-pre; *I*^2^ = 79%) was large, and risk of bias was high. The meta-analysis showed an overall small effect size of 0.26 [0.10, 0.42] in the acquisition phase for participants in the DL group compared to other motor learning methods. In the retention phase, an overall medium effect size of 0.61 [0.30, 0.91] was observed for participants in the DL group compared to other motor learning methods.

**Discussion/Conclusion:** Given the large amount of heterogeneity, limited number of studies, low sample sizes, low statistical power, possible publication bias, and high risk of bias in general, inferences about the effectiveness of DL would be premature. Even though DL shows potential to result in greater average improvements between pre- and post/retention test compared to non-variability-based motor learning methods, more high-quality research is needed before issuing such a statement. For robust comparisons on the relative effectiveness of DL to different variability-based motor learning methods, scarce and inconclusive evidence was found.

## Introduction

Motor learning is a set of processes associated with practice or experience leading to relatively permanent gains in the capability for skilled performance (Schmidt and Lee, 2013). From an applied point of view, the focus of motor learning is on how different practice variables impact performance to lead to relatively permanent changes in capability. Differential learning (DL) is a motor learning method that was proposed in 1999 (Schöllhorn, 1999) and considers learning of a movement or action as being dependent on the amount of noise (practice variability) that accompanies the acquisition process (etiology: *learning from differences*).

Traditional (= non-variability based) motor learning (TL) methods include, for instance, repetitive practice (REP) (Gentile, 1972) or methodological series of exercises (MSE) (Djatschkow, 1973) wherein practice variability is minimized to natural movement variability and a fixed progression of exercises. In contrast, methods such as variable practice (VP) (Schmidt, 1975), contextual interference (CtIt) (Shea and Morgan, 1979), DL (Schöllhorn et al., 2010a), structural learning (SL) (Braun et al., 2010; Hossner et al., 2016b), or the constraint-led approach (CLA) (Renshaw et al., 2010) utilize practice variability in an attempt to further enhance motor learning outcomes. Schöllhorn et al. (2009a) depicted these various motor learning methods in a continuum of increasing variability and noise, with optimal variability levels being dependent on subject and situational constraints (Schöllhorn and Horst, 2020). In practice, however, these different theoretical concepts are often merged when trainers or clinicians aim to improve the motor performance of athletes or patients.

DL distinguishes itself from the other methods in the sense that its rationale is based on the rebuttal of two implicit assumptions in other methods, namely, (1) the to-be-learned movement is considered independent of the individual and time, and (2) the movement performance can be improved by repetitions of (invariant parts of) the movement (Schöllhorn et al., 2010a). In brief, this implies that practicing a movement needs to be done in many varieties and thus no exact repetition, and without corrective feedback on the movement pattern (Hackfort et al., 2019). An example of Peter Valentiner utilizing the DL approach in shot put training can be found online^1^ and implies that the athlete continuously varies the technique used in an attempt to explore movement patterns to discover what works best.

The inspiration for DL's crucial role of practice variability in learning comes from principles of self-organization and dynamical systems theory (Schöllhorn, 2000; Frank et al., 2008) and the concept of stochastic resonance. Although not a central component in the DL theory (Schöllhorn, 2016), the following explanations can be found on the concept of stochastic resonance: “*With an increasing number of offered exercises the probability increases of having one exercise for every group member where s/he will respond to in an adequate way*” (Schöllhorn, 2000). “*By confronting athletes with a high number of practice activities, the probability increases that any of the training exercises can get in resonance with the athlete*'*s needs*” (Schöllhorn et al., 2006). Here, the rationale is for DL exercises to cover a maximal range (or plausible range) of motion patterns in order to maximize the chance that they get in resonance with the individual and time-dependent optimum. In other words, the learner discovers useful components during the exploration of various movement executions that are beneficial for the learner's specific constraints at that time point.

However, the theory and mechanism behind the DL method is not undebated (Schoner, 1995; Scholz and Schöner, 1999; Latash et al., 2007; Beek, 2011; Künzell and Hossner, 2012, 2013; Schmidt and Hennig, 2012; Willimczik, 2013; Schöllhorn et al., 2015; Hossner et al., 2016a; Schöllhorn, 2016). Experimental designs and theoretical rationales of DL have been put forward and discussed but require further examination (Schöllhorn et al., 2009a, 2010a; Schöllhorn and Horst, 2020). The most recent review (Schöllhorn and Horst, 2020) explains DL's enhanced learning rate by an overloading mechanism of the pre-frontal cortex with too many decisions regarding movement execution, which would subsequently enlarge the working memory of the motor control system. There is evidence based on EEG data that suggests DL to cause different brain processes immediately after a training session (Henz and Schöllhorn, 2016; Henz et al., 2018), but in isolation, these data cannot confirm the underlying neural mechanisms of DL and reveal the need for further research.

Regardless of the underlying neural mechanism at play, DL has been experimentally tested in various settings with a large range in the rates of success. The initial experiments were mainly oriented toward performance in a single movement in a sport context (Schöllhorn et al., 2004; Beckmann and Schöllhorn, 2006) or laboratory tasks (James, 2014; James and Conatser, 2014), but recently, it has been adopted within more complex tactical sport contexts (Mateus et al., 2015; Coutinho et al., 2018; Santos et al., 2018), clinical settings (Repšaite et al., 2015; Kurz et al., 2016; Benjaminse et al., 2017; Pabel et al., 2017, 2018; Gokeler et al., 2019), and industrial production processes (Weisner et al., 2019). Collectively, these findings hold valuable information which could support trainers in developing tailored athletic training programs and working toward maximal performance, and could aid clinicians working in injury prevention and rehabilitation.

Despite DL being proposed over 20 years ago, no comprehensive overview with additional analyses currently exists comparing the learning rate of DL with the learning rate of various other motor learning methods. Providing such an overview with analyses could help trainers and clinicians to make better-informed decisions concerning the choice of one or more particular motor learning method(s) in daily practice. However, to date, no systematic review and meta-analysis exists that examines the effectiveness of DL compared to traditional or other variability-based motor learning methods on the performance enhancement of skill (sport context: e.g., dribbling, shooting) or movement tasks (laboratory setting: e.g., unilateral arm rotations) in both the acquisition and retention phase. Therefore, the objective of this meta-analytical review is to examine the evidence from (cluster-)randomized experiments (S) that compared the learning rate of DL (I) to other motor learning methods (C: REP, MSE, VP, CtIt, CLA, and SL) in the performance of movement tasks or skills (O) in humans (P) (PICOS: Population, Intervention, Control, Outcome, Studies). Based on the dynamical systems model of DL by Frank et al. (2008) and the review of Lage et al. (2015), we hypothesized that the learning effectivity of DL would be larger in the retention phase than in the acquisition phase. Besides a systematic summary of the evidence, this meta-analytic review can also be used to explore whether the current empirical evidence supports the claim of DL being an enhanced learning method, to identify gaps in the current state of the art, and to stress various research methodological aspects that require improvement in future research.

## Methodology

The Preferred Reporting Items for Systematic Reviews and Meta-Analyses (PRISMA) statement was followed for the development of the abovementioned research question and review protocol (Moher et al., 2015; Shamseer et al., 2015). The scope of the PICOS question was very broad and consequently stresses the fact that the meta-analysis is rather exploratory in nature. Patterns in the dispersion of results of different studies are as much of interest as the overall mean effects (Borenstein et al., 2009).

### Information Sources

PubMed (MEDLINE), Web of Science, and Google Scholar were searched for relevant articles.

### Eligibility Criteria

The a priori set inclusion criteria were as follows: (1) studies had to be (cluster-)randomized controlled experiments comparing DL to a different motor learning method; (2) the use of co-interventions (e.g., physical literacy and strength training) in both groups was allowed since they represent general practice in non-laboratory contexts and are in line with *representative learning design* directives to ensure functionality and action fidelity in training and learning environments (Pinder et al., 2011); (3) studies had to describe the effects on one or more measures of performance in a movement task; (4) the study report had to be published as a full paper in a journal or as a book chapter to be able to make a reliable risk-of-bias assessment. Exclusion criteria encompassed the following: (1) lack of practice for the control group; (2) the use of non-performance outcomes (e.g., movement patterns), as it is unclear what changes constitute improvement or deterioration, and would be in contradiction with the DL assumptions. In addition, no specific criteria were specified for the population. No restrictions were applied to language or year of publication. DL was defined according to the definition in the Dictionary of Sport Psychology (2019) (Hackfort et al., 2019).

### Search Process

The search strategy was developed by two authors (BS and BT). The following search string was used in PubMed: *[((differential-learning) OR differential-training) OR differencial-learning] OR differencial-training[all]*. The last search was carried out on February 3, 2020. To ensure a sensitive search strategy, additional searches were done based on the reference lists of included articles and reviews, and on the ResearchGate profiles of authors of included articles.

### Screening Procedure

All retrieved titles, abstracts, full texts, and citations were integrated in the Rayyan web application (https://rayyan.qcri.org) (Ouzzani et al., 2016). After removal of duplicates, titles and abstracts were screened, followed by an inspection of the full text. All full texts were independently screened by two authors (BS and BT). In case of disagreement on the eligibility of a study, a third researcher (JV) checked the variable in the original study and agreement was sought by consensus. The following information was extracted: first author, year of publication, study design, description of participants (number, age, gender, and other characteristics), description of the movement task and the performance variable, and description of the training intervention of the DL and other groups (context of the intervention, duration, frequency, number of exercises, number of repetitions, and description of the exercises).

### Risk of Bias Assessment

The included studies were assessed using the Cochrane Risk of Bias Tool, analyzing eight sources of bias: selection, performance, detection, attrition, reporting, and other reasons of bias (Moher et al., 2010). This was done independently by two authors (BS and BT) and discrepancies were resolved through discussion. In case of disagreement, a third researcher (JV) was consulted and agreement was sought by consensus.

### Calculation of Effect Sizes for Quantitative Synthesis

The effect size of choice was a standardized mean difference (Morris, 2008):

$d=\frac{c\times \left[\left(M_{post,DL}-M_{pre,DL}\right)-\left(M_{post,C}-M_{pre,C}\right)\right]}{SD_{pre}}$, where *c* represents a correction factor for small sample sizes (close to 1 for large samples), *M* are means, *SD*_*pre*_ is the pooled standard deviation at the pre-test, and *C* is the control group (other motor learning method). This effect size represents a standardized difference in learning rate between the DL and control group. Learning rate was presented as the order parameter most relevant for DL (Frank et al., 2008). The same effect size was used for the retention test (retention – pre). When a study reported more than one retention test, the latest test was used in our analysis. Results on transfer tests to other than the target movement were not included because there were too few studies on transfer effects. In studies that provided no means and SEs or SDs, but the individual change scores (δ) were given, the effect size was calculated as $d=\frac{c\times \left(M_{\delta ,DL}-M_{\delta ,C}\right)}{SD_{\delta ,pooled}}$. To estimate the standard error of *d*, we needed the pre–post correlation, but this was not included in any report. For the primary analysis, we took *r* = 0.50 as a reasonable mean estimate. Sensitivity analyses were performed with *r* = 0.15 and 0.85 to examine the influence of this parameter on the overall results of the meta-analysis. In case of a discrete outcome measure (e.g., fail or pass on an exam), the log odds ratio was calculated for the data presented in this study and then converted to a standardized mean difference with the formulas presented in Borenstein et al. (2009) (chapters 5 and 7). Similar procedures were applied for studies reporting log odds ratios. For studies that reported multiple outcome variables, we calculated the weighted average effect size. When a study did not report all outcomes, authors were contacted by email. When authors did not respond, but the article contained figures with enough information to calculate the effect size, a software program (GetData-Graph-Digitizer.com) was used to extract the raw study data. However, when authors did not respond and data could not be extracted via other means, the article was excluded from the final quantitative analysis. The interpretation of the effect sizes was done in accordance with Cohen's (1988) guidelines: “negligible,” *d* < 0.2; “small,” 0.2 < *d* < 0.5; “medium,” 0.2 < *d* < 0.8; “large,” *d* > 0.8 (Cohen, 1988).

### Meta-Analyses

Separate meta-analyses for the effects of acquisition (pre-test vs. post-test) and learning (pre-test vs. retention test) were carried out. Subgroup analyses were performed based on the type of task (e.g., sport performance, technical skill) and type of contrasted learning method (e.g., DL vs. TL and DL vs. CtIt). Subgroups based on the type of task were defined by the following separation criteria: (1) “sport performance” encompassed outcomes focusing on the speed or strength component of the skill performed by the participant. For example, how far a participant could throw, how fast a participant sprinted in a straight line or around the track, how high a participant jumped, how hard a participant could kick a ball, etc. (i.e., shot put, high jump, hurdle racing, ice skating race, and countermovement jump); (2) “sport technical skills” focused more on the precision aspect of skills (e.g., shooting/passing/kicking/serving accuracy as measured by the error with respect to a target, reception of a pass as measured by the distance from the reception point, completion of a technical/agility circuit against time); (3) “sport tactical behavior (skills)” included outcomes assessed during match play (e.g., triple threat position/give-and-go/explore 1-on-1 game/field goals characterized as whether the behavior was successful or not; these variables were then normalized); (4) “fine motor skills”: healthy participants had to carry subtle or refined movement tasks or skills outside the sport context (i.e., toothbrushing, dental surgery, handle rotation, and standing as still as possible); (5) “rehabilitation”: injured or post-operative participants (this category was left out of the meta-analysis, since the two studies could not be included in the quantitative analyses). All meta-analyses were carried out in Review Manager 5.3 (Cochrane Collaboration). Studies that used different subgroups (e.g., based on age) were entered separately in the meta-analysis. Random effects models were used throughout as between-study variation was expected based on the heterogeneity of movement tasks, subject characteristics, study designs, and performance variables (Borenstein et al., 2009). The inverse of the variance was used to weigh each study result on the overall mean and 95% CI. For the interpretation of heterogeneity, Higgins' *I*^2^ values were calculated (Higgins et al., 2003). Publication bias was visually inspected with a funnel plot. Supplementary material may be found online at https://osf.io/m4sje/.

## Results

### Qualitative Synthesis

**Figure 1:**
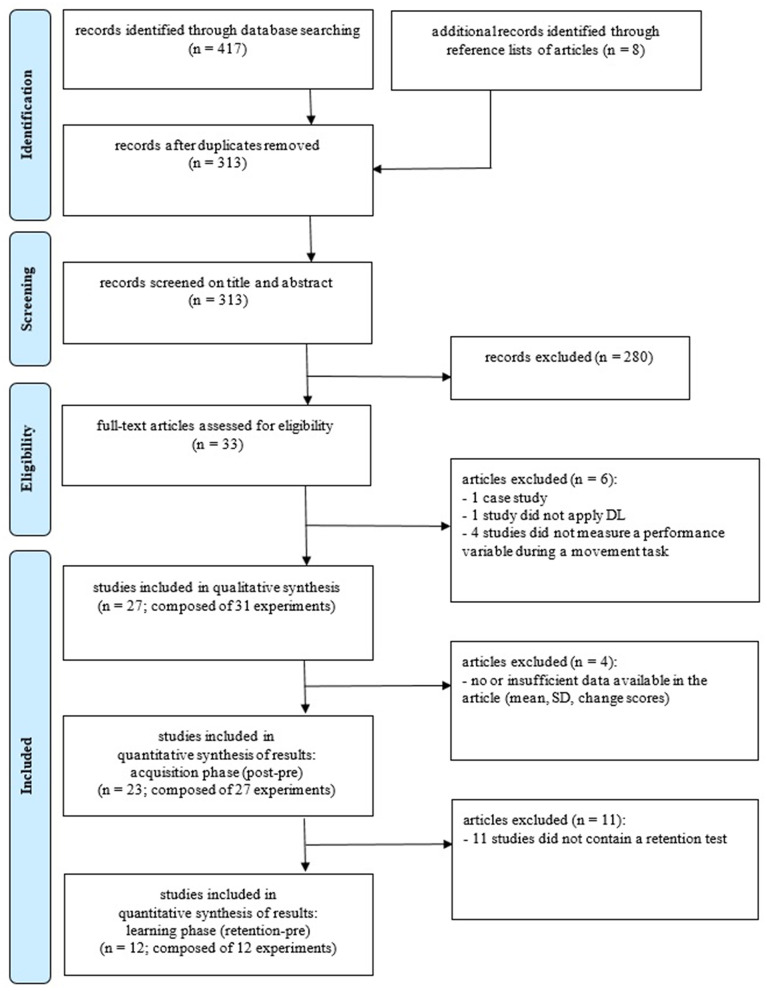
Flowchart of the search and screening process (based on the PRISMA statement template). DL, differential learning; TL, traditional learning; CtIt, contextual interference; SL, structural learning (Moher et al., 2015).

The flowchart in Figure 1 shows the results of the search and screening process, as well as the numbers of articles included. For the qualitative synthesis, there are 27 original studies included that contain 31 original experiments. For the quantitative synthesis (acquisition phase), there are 23 original studies included that contain 27 original experiments. For the quantitative synthesis (learning phase), there are 12 original studies included that contain 12 original experiments. The features of the included articles are described in Table 1.

**Table 1:** Design, participants, movement tasks, performance variables, and training interventions of studies included in the qualitative synthesis.

**First author, year, design**	**Participants**	**Context**	**Movement task**	**Performance variable**	**Duration, frequency**	**Differential learning**	**Other training**
Schöllhorn et al. (2004) (exp. 1) PPC: DL vs. TL	Trained soccer players (M) in the German regional league (age 21.9 ± 3.7) DL: *n* = 10 TL: *n* = 10	Supplemental to normal club training	Soccer: goal shooting (sport technical skills)	Points scored over 35 trials divided over 4 initial ball locations (optimal target locations received more points)	6 weeks 2 sessions week^−1^ (25 min)	nr. of exercises: ? nr. of repetitions: 1 exercises described: no, only sources of variation feedback: no	TL: REP nr. of repetitions: ? reference of optimal motion: yes feedback: yes, corrective instructions
Schöllhorn et al. (2004) (exp. 2) PPC: DL vs. TL	Trained soccer players (M) from a senior (age 23.5 ± 3.8) and a junior (12.1 ± 1.7) soccer team. DL: *n* = 8 senior + 14 junior TL: *n* = 8 senior + 13 junior	Supplemental to normal club training	Soccer: dribbling and passing	Passing the ball toward a target at 20 m in front of the subjects. Straight pass 6 points, less points for deviations to the left and right. Task was performed 5 times.	4 weeks 3 sessions week^−1^ (20–40 min)	nr. of exercises: ? nr. of repetitions: 1 exercises described: no feedback: no	TL: REP nr. of repetitions: ? reference of optimal motion: yes feedback: yes, corrective instructions
Schöllhorn et al. (2004) (exp. 3) PPC: DL vs. TL	Soccer players from the German provincial and regional leagues. DL: *n* = 12 (mean age 23.8) TL: *n* = 13 (mean age 28.1)	Supplemental to normal club training	Soccer: ball reception test	Distance between initial ball contact and the position of the ball after control when receiving the ball.	4 weeks 7 sessions of 15–20 min	nr. of exercises: 18–24 per session nr. of repetitions: 1 exercises described: no feedback: ?	TL: REP nr. of repetitions: ? reference of optimal motion: yes feedback: yes, corrective instructions
Schöllhorn et al. (2006) (exp. 1) PPC: DL vs. TL	Senior soccer team 5th German division (M). Allocation based on pre-test scores. DL: *n* = 8 TL: *n* = 8	Supplemental to normal club training	Soccer: dribbling and passing	Passing the ball toward a target at 20 m in front of the subjects. Straight pass 6 points, less points for deviations to the left and right. Task was performed 5 times.	4 weeks 3 sessions week^−1^ (20–40 min)	nr. of exercises: ? nr. of repetitions: 1 exercises described: no feedback: no	TL: REP nr. of repetitions: ? reference of optimal motion: yes feedback: yes, subjects received a detailed description of ideal pattern and corrective instructions
Schöllhorn et al. (2006) (exp. 2) PPRC: DL vs. TL	Players from the 5th and 7th German national soccer division (M). Allocation based on pre-test scores. DL: *n* = 9 TL: *n* = 9	Supplemental to normal club training	Soccer: goal shooting	Points scored over 35 trials divided over 7 initial ball locations (optimal target locations received more points)	6 weeks 2 sessions week^−1^ (25 min, no goal shooting during regular training) retention test: 1 year after post-test	nr. of exercises: ? nr. of repetitions: 1 exercises described: no, only sources of variation feedback: ?	TL: MSE nr. of repetitions: 5–10 per exercise feedback after every shot: error descriptions, movement-oriented corrections, metaphoric instructions
Beckmann and Schöllhorn (2006) PPRC: DL vs. TL	Sports science students (12 M + 12 F, age 22.1 ± 3.8). No experience in shot put. Allocation to groups was based on pre-test scores. DL: *n* = 12 (6M + 6F) TL: *n* = 12 (6M + 6F)	University sports class	Shot put (mass of the shot: F = 3, 4 kg, M = 6.25 k)	The average shot distance of three trials. Sufficient recovery time between trials.	4 weeks 2 sessions week^−1^ (60 min) retention tests: 2 and 4 weeks after post-test	nr. of exercises: ± 30 per session nr. of repetitions: 1 exercises described: no, only sources of variation feedback: no	TL: MSE nr. of repetitions: 10–15 per exercise reference of optimal motion: yes feedback: yes, corrective instructions
Torrents et al. (2007) Longitudinal follow-up:	Two female national standard aerobic gymnasts (age 20 and 21)	Integrated during regular training sessions	(1)One-armed push-ups: right (2) One-armed push-ups: left (3) Hinge push-ups (4) Leap jump (5) Straddle jump (6) Half turn straddle jump	Absolute time of execution to complete push-up as fast as possible within 4 s. Flight time of each jump. Each test was repeated 3 times and the best time was analyzed.	18 weeks 6 sessions week^−1^ (3 h): - 5 weeks TL - 8 weeks DL - 5 weeks TL Performance was evaluated weekly by means of 6 tests	nr. of exercises: ? nr. of repetitions: ? exercises described: ? feedback: ?	TL: MSE nr. of repetitions: ? reference of optimal motion: yes feedback: ?
Schöllhorn et al. (2008) PPRC: DL vs. TL	3 F + 9 M well-trained tennis players (tennis experience: between 17 and 34 years in regional tennis league). Allocation to groups was based on pre-test scores. DL: *n* = 6 TL: *n* = 6	Supplemental to normal club training	Tennis service	3 × 4 services from the left and right side toward different target zones. According to the tactical advantage of each zone, the service received 1/2/3/4 points. Sum of the points is the performance variable.	6 weeks 2 sessions week^−1^ retention test: 2 weeks after intervention	nr. of exercises: ± 90 services per session nr. of repetitions: ? exercises described: no, only sources of variation feedback: no	TL: MSE nr. of exercises: ± 90 services per session nr. of repetitions: ? reference of optimal motion: yes feedback: yes, corrective instructions
Schöllhorn et al. (2009a) PPRC: DL vs. TL	36 M, 21 F novice high jumpers, age 22.8 ± 2.2. Allocation was based on the results of the pre-test. DL: *n* = 19 TL: *n* = 19	?	Fosbury flop and jump and reach test.	Best performance of two trials (maximal height)	4 weeks 2 sessions week^−1^ retention: 10 days after post-test	nr. of exercises: ? nr. of repetitions: 1 exercises described: no, only sources of variation feedback: no	TL: MSE nr. of exercises: ? nr. of repetitions: ? reference of optimal motion: yes feedback: yes, corrective instructions
Schöllhorn et al. (2010b) PPC: DL vs. TL	Athletic club athletes, age 13.2 ± 1.7. DL: *n* = 15 TL: *n* = 13	Supplemental to normal club training	60 m hurdle race	Time to finish (measured with light barriers)	6 weeks 4 sessions week^−1^ (90 min of which 30 min for hurdle training)	nr. of exercises: ? nr. of repetitions: 1 exercises described: no, only sources of variation gradual DL: every exercise was combined with a new instruction that was related to the previous exercise, but with an additional task. feedback: no	TL: MSE nr. of exercises: ? nr. of repetitions: 3 per exercise reference of optimal motion: yes feedback: yes, corrective instructions and explanations about technique of world class athletes through video and photographs.
Beckmann et al. (2010) PPRC: DL1 vs. DL2 vs. DL3 vs. CtIt	Experienced hockey players. DL1: *n* = 9 DL2: *n* = 9 DL3: *n* = 9 CtIt: *n* = 9	Supplemental to normal club training	Hockey: push and flick toward goal (targets bottom right and top left, respectively).	Target precision (measured with an optic measurement system)	6 weeks 2 sessions week^−1^ retention: 2 and 4 weeks after post-test	nr. of exercises: 20 for push and 20 for flick DL1: targets were varied in randomized order and no targets were aimed twice. DL2: no target variations, but movement variations DL3: combination of DL1 and DL2 nr. of repetitions: 1 exercises described: no feedback: no	CtIt (no variations, but subjects practiced the push and flick in randomized order) nr. of repetitions: 20 for push and 20 for flick reference of optimal motion: no
Savelsbergh et al. (2010) PPC: DL vs. TL	Adult recreational ice skaters (M), age 44.2 ± 9.8 with 100-m time > 13 s. Allocation to groups was based on pre-test scores. DL: *n* = 9 TL: *n* = 9	Supplemental to normal club training	Ice skating start in a straight line from a stand still position.	Split times were taken at 5, 10, 25, and 49 m. Five trials were performed in a 1-h period.	1 week 3 sessions of 60 min	nr. of exercises: 14 (different start positions) nr. of repetitions: 1 exercises described: yes feedback: no	TL: REP nr. of repetitions: 14 feedback: yes, corrective instructions on starting position reference of optimal motion: yes
Schöllhorn et al. (2012) PPRC: Dlr vs. Dlb vs. TL	8th division of German soccer league. DLr: *n* = 4 (age 24.5 ± 2.1, soccer experience 20.5 ± 1.0) DLb: *n* = 4 (age 24.5 ± 2.1, soccer experience 20.8 ± 3.4) TL : *n* = 4 (age 23.8 ± 3.9) soccer experience 18.5 ± 4.7)	Supplemental to normal club training	Soccer: ball control test and goal shooting test.	Distance between initial ball contact and the position of the ball after control when receiving the ball. Points scored over 35 trials divided over 7 initial locations (optimal targets received more points).	4 weeks 2 sessions week^−1^	nr. of exercises: 20 exercises on ball control and 20 on goal shooting per session nr. of repetitions: 1 exercises described: yes feedback: no DLr: random changes between exercises for ball control and goal shooting DLb: blocked sequence of exercises for ball control and goal shooting	TL: REP nr. of repetitions: 20 repetitions of ball control and 20 of goal shooting per session reference of optimal motion: yes
Reynoso et al. (2013) PPRC: DL vs. TL	Students with no volleyball experience. 11 F, 21 M DL: *n* = 10 (age 21.0 ± 0.94) TL: *n* = 11 (age 22.0 ± 2.10) Before the pre-test, all subjects received an audio-visual introduction to the correct execution of the service (reference to guidelines provided).	?	Volleyball service test. 4 sets of 8 services to a specified target.	Speed and accuracy of the service (measured with radar gun and video camera).	3 weeks 11 sessions	nr. of exercises: 3 sets of 15 exercises per session nr. of repetitions: 1 exercises described: no feedback: in the first two sessions audio-visual information was supported with verbal info when the subjects requested it.	TL: REP nr. of repetitions: 3 sets of 15 repetitions per session feedback: no
James, 2014 PPC: DL vs. TL	14 M, 19 F (age 25.2 ± 4.2) DL: *n* = 16 REP: *n* = 17	Laboratory experiment	Standing as still as possible on one/two legs with eyes open, looking at a dot on the wall.	RMSJ of the head and CoM in AP and ML directions	1 session pre-test, training and post-test on 1 day (15 min seated rest between training and post-test).	nr. of exercises 15 postural training trials of 1 min duration with 30s rest between trials exercises described: yes	TL: REP nr. of repetitions: 15 postural training trials that repeated the 2-leg stance task.
James and Conatser, 2014 PPRC: DL vs. TL	12 M, 15 F (age 23.9 ± 3.8) DL: *n* = 13 REP: *n* = 14	Laboratory experiment	Rotations of a handle (180°) with extended elbows by radioulnar and shoulder in/external rotations. Goal was to make smooth movements to the beat of a metronome (1 and 2 Hz).	RMSJ of the hand during the movement	2 weeks 2 sessions week^−1^ post-test: 24h after last training, retention-test: 2 weeks after post-test 20 practice trials of 1 min per session (1 min rest between trials).	nr. of exercises: 20 per sessions (trials of 1 min, 1 min rest between, self-selected pace and range of motion) nr. of repetitions: 1 exercises described: yes feedback: no	TL: REP nr. of repetitions: 20 per sessions (trials of 1 min, 1 min rest between, self-selected pace and range of motion) feedback: no (but they received the smoothness instruction each session)
Repšaite et al. (2015) PPC: mixed DL-OT vs. OT	Patients that had suffered a cerebral infarction in the left hemisphere who followed occupational therapy courses. 9 M, 18 F (age 73.9 ± 7.7) mixed DL-OT: *n* = 12 OT: *n* = 15	Physical medicine and rehabilitation department (hospital), 10–14 days after stroke onset.	Wolf motor function test which includes 15 functional tasks that have to be completed within 120 s.	Time on each of the tests.	32 days 5 sessions week^−1^ (30 min). Both groups received the same co-interventions.	mixed DL-OT 3 sessions OT week^−1^ and 2 sessions DL week^−1^ modified tools of OT, no specific descriptions included of the variations	TL: OT, exercises and tools for strengthening upper limb muscles, range-of-motion, fine motor skills and coordination nr. of repetitions: ?
Mateus et al. (2015) PPC: DL vs. TL	Physical education students (age 20.4 ± 1.9). DL: *n* = 38 TL: *n* = 38	University sports class	Basketball: technical skills (agility test and taco bell challenge) and tactical skills (4v4 small sided game).	Technical skills: total time to conduct the tests. Tactical skills were assessed with a 4-a-side game (video recording): (un)successful attempts were counted for 4 actions (triple threat position, field goals, give-and-go, explore-1-on-1 game).	8 weeks 2 sessions week^−1^ (120 min) warm-up, small sided games and 5-a-side basketball games within each session was the same for both groups.	nr. of exercises: ? nr. of repetitions: ? exercises described: no feedback: ?	TL: REP nr. of repetitions: ? feedback: ? reference of optimal motion: no
Kurz et al. (2016) PPC: DL vs. TL	Patients after a knee (*n* = 15) or hip (*n* = 11) replacement surgery (age 65.7 ± 9.9). All patients needed to be able to bear their full weight. DL: *n* = 14 TL: *n* = 12	Patients in a rehabilitation center for gait training.	(1) timed up-and-go test (2) 4- and 10-m run test (3) 6-min run test (4) one-leg standing test with eyes open/closed. The transfer test was a variation of (1)	(1) time to complete (2) time to complete (3) distance covered (4) time subject could stand on one leg	3 exercise sessions of 25 min between pre- and post-test	nr. of exercises: ? nr. of repetitions: 1 exercises described: few examples and sources of variation are given feedback: no	TL: REP nr. of repetitions: ? feedback: no reference of optimal motion: no, but demonstrations by physiotherapist were given
Hossner et al. (2016b) (exp. 1) PPC: DL vs. DL+FB vs. TL	Players (M) from a Swiss soccer club. Allocation based on pre-test scores, age and soccer experience. DL: *n* = 10 DL+FB: *n* = 9 TL : *n* = 9	Supplemental to normal club training.	Soccer: 16 goal shots (8 shots from a left and right position subdivided into 4 shots each to a target in the left and right corner of the goal (red disks, 0.2 m diameter).	Shots were filmed: average radial error to target center.	6 weeks 2 sessions week^−1^ (30 min) post-test: 1 week after last session absent sessions: 0.9 ± 1.1 (no difference across groups).	DL: nr. of exercises: 30–35 nr. of repetitions: ? exercises described: no, only sources of variation (initial only 1 source of variation, later combinations were used) feedback: no DL+FB: same as DL with individual feedback when non-optimal performance was noticed that could not be attributed to the current task variant. Augmented feedback was also given to the whole group.	TL: MSE 30–35 shots per session nr. of repetitions per exercise: ? feedback: yes reference of optimal motion: yes
Hossner et al. (2016b) (exp. 2) PPRC: DL vs. SL vs. TL	Sports science students (13 F, 23 M). Allocation based on pre-test score, age, height, sex, shot-put experience, motivation to take part in the study. DL: *n* = 12 TL: *n* = 12 SL: *n* = 12	University sports, students received credits.	Shot put (mass of the shot: F = 4 kg, M = 6.25 kg)	Average distance of 3 shots (sufficient recovery time between trials)	4 weeks 2 sessions week^−1^ (consecutive days) absent sessions: 0.7 ± 0.7 (no difference across groups). Post-test during last session, retention: 2 and 4 weeks after last session	nr. of exercises: 32 per session (last session: 20) exercises described: no, only sources of variation, 2 sources combined per practice trial (random order) Exercises were explained with illustrations nr. of repetitions: 1 feedback: no	TL: MSE nr. of exercises: ? nr. of repetitions: ? (32 trials in total, last session: 20) reference of optimal motion: yes feedback: yes SL: same practice variants as DL, only the order of the variants was different: the sequence of variants was determined in order to minimize the difference between subsequent variants.
Pabel et al. (2017) CRT-PO: DL vs. TL	Third-year students in a preclinical course in operative dentistry (Germany). Both groups had the same laboratory, but no clinical experience. DL: *n* = 32 TL: *n* = 41	University course on operative dentistry.	Preparation of a gold partial crown (dentistry) on training models of the upper and lower jaw fixed in phantom heads.	The exam consisted of preparing a gold crown on tooth 46 within 90 min. Four examiners evaluated the preparation anonymously and independent. Criteria for exam failure are indicated. Pass/fail was the performance variable.	4 days 4 hours training per day	All subjects viewed a video demo with verbal explanations before the training. nr. of exercises: 5 day^−1^ nr. of repetitions: 30 min per exercise exercises described: yes feedback: no	All subjects viewed a video demo with verbal explanations and received demonstration models of an “ideal” preparation and assessment criteria: the ideal dimensions and parameters. TL: MSE nr. of exercises: ? nr. of repetitions: ? feedback: yes (oral and written)
Santos et al. (2017) PPC DL vs. TL	Seventy-six college students in physical education (age = 20.4 ± 1.9 years): *Non-structured path* (*n* = 14) DL: *n* = 6 TL: *n* = 8 *Early specialization* (*n* = 34) DL: *n* = 19 TL: *n* = 15 *Late specialization* (*n* = 28) DL: *n* = 13 TL: *n* = 15	University sports class	Basketball: technical skills (agility test and taco bell challenge) and tactical skills (4v4 full-court basketball game).	Technical skills: total time to conduct the tests. Tactical skills were assessed with a 4-a-side game (video recording): (un)successful attempts were counted for 4 actions (triple threat position, field goals, pass-and-cut, explore-1-on-1 game).	8 weeks in total; 16 classes; two practical classes per week (120 min/class).	*TL group:* nr. of exercises: 7 session^−1^ nr. of repetitions: 45 min in total exercises described: yes feedback: ? *DL group:* nr. of exercises: 30 session^−1^ nr. of repetitions: 45 min in total exercises described: yes feedback: ? *Both groups (DL vs. TL):* Warm-up (10 min) Small-sided games (30 min) Basketball game (15 min)	TL: REP nr. of repetitions: ? feedback: ? reference of optimal motion: no
Pabel et al. (2018) PPRC: DL vs. TL	Children 6–9 years from 1 school (Germany). Allocation was stratified on first/second grade. DL: *n* = 18 TL: *n* = 18	School-based intervention: during lunch break at the school's washrooms.	Tooth brushing	Evaluated by a blinded examiner on two parameters: gingival inflammation (PBI) and plaque scores (T-QHI).	15 working days (3 intervals of 2 days each).	All children were given a toothbrush (changed every 21 days), no other oral hygiene products were allowed (brushing at home could not be controlled). Initial verbal instruction and demonstration on a model. nr. of exercises: 15 (1 per day) nr. of repetitions: 3 min exercises described: yes feedback: no	TL: REP All children were given a toothbrush (changed every 21 days), no other oral hygiene products were allowed (brushing at home could not be controlled). Initial verbal instruction and demonstration on a model. nr. of repetitions: 3 min reference to optimal motion: yes feedback: yes
Santos et al. (2018) PPC: DL vs. TL	Portuguese youth soccer players (two different U13 and U15 teams at regional level). DL-U13: *n* = 10 (age 11.1 ± 0.5, experience 4.4 ± 2.9) DL-U15: *n* = 10 (age 13.1 ± 0.3, experience 7.1 ± 1.5) TL-U13: *n* = 10 (age 11.4 ± 0.5, experience 5.3 ± 2.5) TL-U15: *n* = 10 (age 13.0 ± 0.8, experience 6.8 ± 1.6)	Supplemental to normal club training.	Soccer: 5 vs. 5 small sided game, 2 bouts of 6 min (3 min rest between)	Games were recorded and behavior was assessed with notational analysis. Fails, attempts, fluency, versatility and originality occurrences were recorded for passes, dribbles and shots.	5 months 3 sessions week^−1^ (30 min before the regular club training)	nr. of exercises: ? nr. of repetitions: ? exercises described: yes (sources of variation and many examples of each) feedback: no	TL: small-sided-games with fewer variations than DL nr. of repetitions: ? feedback: ?
Coutinho et al. (2018) CRPP: DL vs. TL	Portuguese youth soccer players (attackers only) from two teams. DL-U15: *n* = 9 (age 14.2 ± 0.8, experience 6.4 ± 3.2) DL-U17: *n* = 6 (age ?, experience 6.4 ± 3.2) TL-U15: *n* = 9 (age 13.9 ± 0.5, experience 6.1 ± 3.1) TL-U17: *n* = 6 (age 16.1 ± 0.7, experience 8.0 ± 2.1)	Supplemental to normal club training.	Soccer: technical skills (vertical jump, speed, agility), and tactical behavior [5 vs. 5 small sided game, 3 bouts of 6 min (3 min rest)]	Vertical jump: counter movement. Speed: 30-m sprint test. Agility: repeated change of direction task: 6 × 20 m sprints with 4 100° change of direction (optical timing system used for all tests). Games were recorded and assessed with notational analysis. Fails, attempts, fluency, versatility and originality occurrences for passes, dribbles, and shots.	10 weeks 2 sessions week^−1^ (25 min intervention + 65 min regular training) intervention: 10 min physical literacy + 15 min small-sided games	nr. of exercises: ? nr. of repetitions: ? exercises described: yes (sources of variation and many examples of each) feedback: no	TL: regular club training nr. of exercises: ? nr. of repetitions: ? feedback: ?
Bozkurt, 2018 PPC: DL vs. TL	Turkish soccer players (U15 team) DL: *n* = 6 TL: *n* = 6	Supplemental to normal club training.	Soccer: technical skills test battery	Passing: Mor-Christian soccer passing test, German Football Association agility and dribbling test, feet juggling test.	4 weeks 3 sessions week^−1^ 8/12 players attended the full program	nr. of exercises: 9 exercises for target-passing, 9 for dribbling and 9 for feet-juggling techniques (blocked order) nr. of repetitions: ? exercises described: no (only sources of variation) feedback: no	TL: MSE nr. of exercises: 9 exercises for target-passing, 9 for dribbling and 9 for feet-juggling techniques (blocked order) reference to optimal motions: no nr. of repetitions: ? feedback: yes
Weisner et al. (2019) PPRC: DL vs. TL	Assembly line workers. DL: *n* = 11 (4F, age 22–64, median experience 2) TL: *n* = 11 (4F, age 21–61, median experience 3)	Field study: industrial engineering training center (Institute of Production Systems, Dortmund)	Production of a 2-speed-gearbox in 6 assembly cycles.	Assembly cycle times and assembly errors (test duration *n* = 60 min).	3 weeks 5 sessions total (60 min session^−1^)	nr. of products: 28 nr. of exercises: ? exercises described: no (only sources of variation) feedback: no	TL: REFA-Work instructions (based on optimal pattern) feedback: yes
Gaspar et al. (2019) PPC: DL vs. TL	Portuguese soccer players (U15) with at least 2 years of soccer-specific training experience DL: *n* = 20 TL: *n* = 20	Integrated during regular training sessions	Soccer kicking performance and countermovement jump	Kicking task: (1) Ball velocity (2) Ball speed (3) Accuracy Jump height	1 day 1 training session: 36 repetitions from the same 3 kicking locations with 18 different kicking variations. Each variation was completed from kicking a static ball and after a 5-m dribble	*DL* nr. of exercises: 18 nr. of repetitions: 2 exercises described: yes (sources of variation and many examples of each) feedback: no	TL: MSE nr. of exercises: 6 exercises for static ball kicking after 5-m run up, 6 exercises for ball kicking after a 5-m dribble. reference to optimal motions: yes nr. of repetitions: 6 feedback: yes
Serrien et al. (2019) PPRC DL vs. CtIt	Students or teaching/research assistants in physical education, movement science, physiotherapy or manual therapy: DL: *n* = 16 (3F; age = 24 ± 2 years; exercise/week = 4 ± 1 h) CtIt *n* = 16 (4F; age = 23 ± 2 years; exercise/week = 4 ± 1 h)	Laboratory experiment	Goalkeeping mimicking task	Visuomotor reaction time: extinguish LED-lights placed on a wall as fast as possible.	1 day 1 training session: 180 stimuli for both DL and CtIt group (± 30 min) Post-test immediately after training session; Retention-test: same day, after 1 h of rest	*DL* nr. of exercises: 30 nr. of repetitions: 6 exercises described: yes feedback: mean response time and number of missed targets during warmup	CtIt: blocked reference of optimal motion: no nr. of exercises: 3 nr. of repetitions: 2 × 30 exercises described: yes feedback: mean response time and number of missed targets during warmup
Ozuak and Çaglayan (2019) PPC: DL vs. TL	Turkish soccer players (age 11–13) DL: *n* = 26 TL: *n* = 26	Supplemental to normal club training.	(1) Illinois Agility Test (2) Creative Speed Test (3) Ball Dribbling Test (4) Ball Juggling Test (5) Passing Test	(1) time to complete (2) time to complete (3) time to complete (4) nr. of times they keep the ball in the air while juggling (5) number of passes (out of 12) that reached the target	8 weeks, 3 sessions week^−1^, (40–50 min session^−1^), after which, the participants continued with soccer training	nr. of exercises: 14 nr. of repetitions: 1 exercises described: yes feedback: no	TL: regular club training nr. of exercises: ? nr. of repetitions: ? feedback: ?

*exp, experiment; PPC, pre-test–post-test design with control group; PPRC, pre-test–post-test design with retention test and control group; CRT-PO, cluster-randomized trial post-test only; CRPP, cluster-randomized pre-test–post-test design; M, male; F, female; DL, differential learning; TL, traditional learning (REP and MSE); REP, repetitive practice; CtIt, contextual interference; SL, structural learning; RMSJ, root-mean-square-jerk; CoM, center-of-mass; AP, antero-posterior; ML, media-lateral; MSE, methodological series of exercises; OT, occupational therapy; ?, not described in the article/chapter or only generic statements regarding the content*.

Twenty-seven articles met the inclusion criteria, resulting in 31 experiments providing data on 897 participants (DL group: *n* = 453; control group: *n* = 446). DL has been used in a variety of contexts: (1) sport performance outcomes (i.e., shot put, high jump, hurdle racing, ice skating race, and countermovement jump); (2) technical skills in a single sports movement (i.e., service in volleyball/tennis; soccer: passing, shooting accuracy, and ball control; hockey: goal shooting precision); (3) tactical skills in a sport context (i.e., during match play in basketball or soccer); (4) fine motor skills (toothbrushing, dental surgery, handle rotation, and balance); and (5) rehabilitation (Repšaite et al., 2015; Kurz et al., 2016). Mateus et al. (2015), Santos et al. (2017), and Coutinho et al. (2018) assessed the effects of DL on both technical and tactical skills. The majority of studies examined the effects of DL directly after the intervention (acquisition effect), while only 12/27 experiments included a retention test (learning effect). When available in the manuscript, Table 1 summarizes the timing of post- and retention tests and delays between them. Most post-tests were organized on the same day or within 24 h of the last training session whereas some post-tests were organized a week after the last training session. The time between post-test and retention test varied between 1 h and 1 year (most studies between 1 and 2 weeks).

### Risk of Bias Analysis

Table 2 gives an overview of the risk of bias of each study (experiment). Concerning randomization, 15/31 experiments had a low risk of bias and the other were unclear, whereas two studies used cluster randomization (high risk). Allocation concealment was unclear in all but four experiments with high risk of bias and two with low risk of bias. Given the nature of the experiments, blinding of participants and personnel was not possible. Outcome assessment was blinded in 7/31 experiments and unclear otherwise (blinded researcher or computerized registrations). Incomplete outcome data were high risk or unclear in 8/31 experiments, the rest had low risk. Selective outcome reporting was high risk of bias in 9/31 experiments (reported no means, standard deviations, and/or statistics and did not respond to emails for further inquiry). Other reasons of bias were an incomplete description of the training/control intervention and outcome variables that are susceptible to subjective interpretation. With exception for the studies from the groups of Savelsbergh, James, Hossner, Pabel, and Serrien, risk of bias was overall high for all studies (fewer than 4/7 items with low risk of bias).

**Table 2:** Risk of bias analysis.

**References**	**A**	**B**	**C**	**D**	**E**	**F**	**G**	
Schöllhorn et al. (2004) (exp. 1)	+	?	–	?	?	–	–	^*^
Schöllhorn et al. (2004) (exp. 2)	+	?	–	?	?	–	–	^*^
Schöllhorn et al. (2004) (exp. 3)	+	?	–	?	?	–	–	^*^
Schöllhorn et al. (2006) (exp. 1)	?	?	–	?	+	+	–	^*^
Schöllhorn et al. (2006) (exp. 2)	+	?	–	?	+	+	–	^*^
Beckmann and Schöllhorn (2006)	+	?	–	?	+	+	–	^*^
Torrents et al. (2007)	–	–	–	–	?	+	?	
Schöllhorn et al. (2008)	+	?	–	?	+	–	–	^*^
Schöllhorn et al. (2009b)	+	?	–	?	+	–	–	^*^
Schöllhorn et al. (2010b)	+	?	–	?	+	–	+	^*^
Beckmann et al. (2010)	+	?	–	+	+	–	–	^*^
Savelsbergh et al. (2010)	+	?	–	+	?	+	+	^*^
Schöllhorn et al. (2012)	?	?	–	?	–	+	+	^*^
Reynoso et al. (2013)	+	?	–	?	+	+	–	^*^
James (2014)	?	?	–	+	+	+	+	^*^
James and Conatser (2014)	?	?	–	+	+	+	+	^*^
Mateus et al. (2015)	?	?	–	?	+	+	–	^*^
Repšaite et al. (2015)	?	?	–	+	+	–	–	
Kurz et al. (2016)	?	?	–	–	–	+	–	
Hossner et al. (2016b) (exp. 1)	+	?	–	?	+	+	+	^*^
Hossner et al. (2016b) (exp. 2)	+	?	–	?	+	+	+	^*^
Pabel et al. (2017)	–	–	–	+	+	+	+	^*^
Santos et al. (2017)	?	+	?	?	+	+	–	^*^
Pabel et al. (2018)	+	?	–	+	+	+	+	^*^
Bozkurt (2018)	?	?	–	?	–	+	–	^*^
Santos et al. (2018)	?	?	–	?	+	+	–	^*^
Coutinho et al. (2018)	–	–	–	?	+	+	–	^*^
Weisner et al. (2019)	?	?	–	?	+	–	–	
Gaspar et al. (2019)	–	–	–	?	+	+	+	^*^
Serrien et al. (2019)	+	+	–	?	+	+	+	^*^
Ozuak and Çaglayan (2019)	?	?	–	?	+	+	–	^*^

A, random sequence generation; B, allocation concealment; C, blinding of participants and personnel; D, blinding of outcome assessment; E, incomplete outcome data; F, selective reporting; G, other bias; ^*^, study included in meta-analysis; +, low risk; ?, unclear risk; –, high risk.

### Quantitative Synthesis of Results

**Figure 2:**
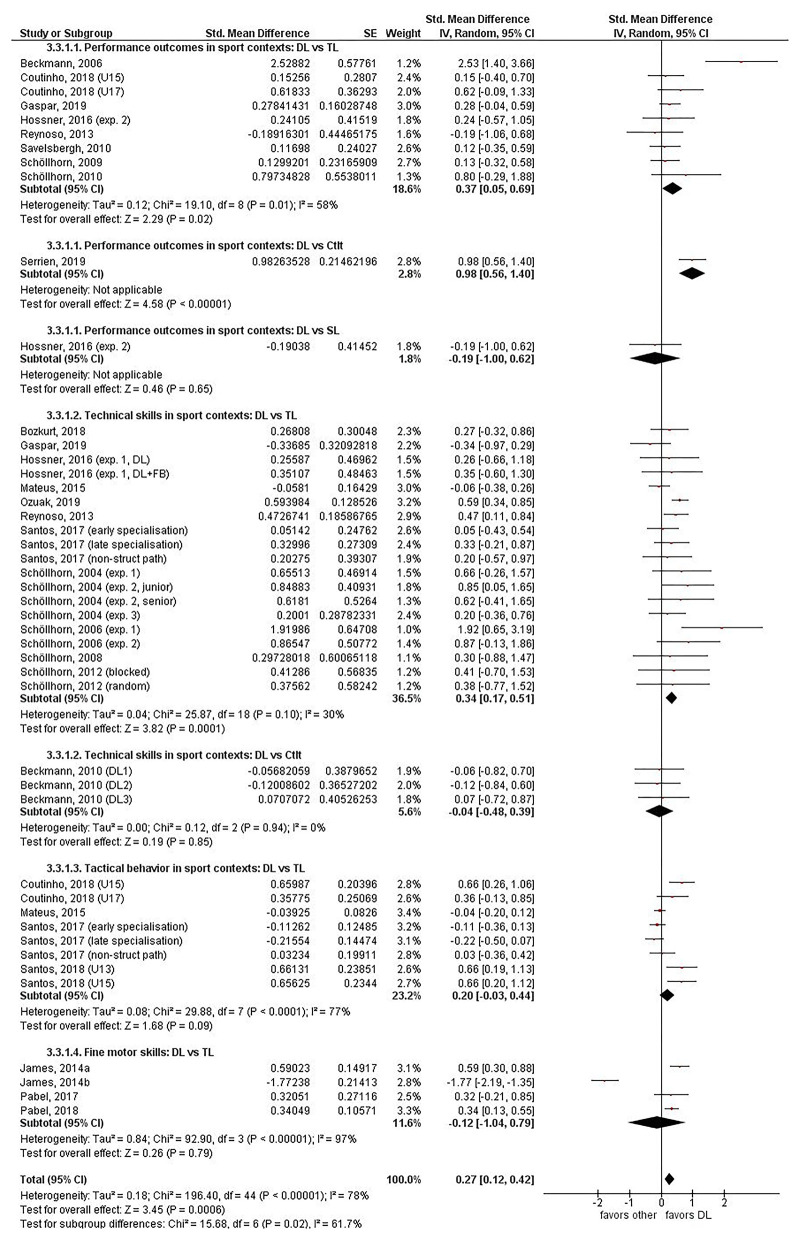
Acquisition phase (post – pre). Forest plot for the effects of differential learning vs. other methods grouped by category of movement task. DL, differential learning; TL, traditional learning; CtIt, contextual interference; SL, structural learning.

**Figure 3:**
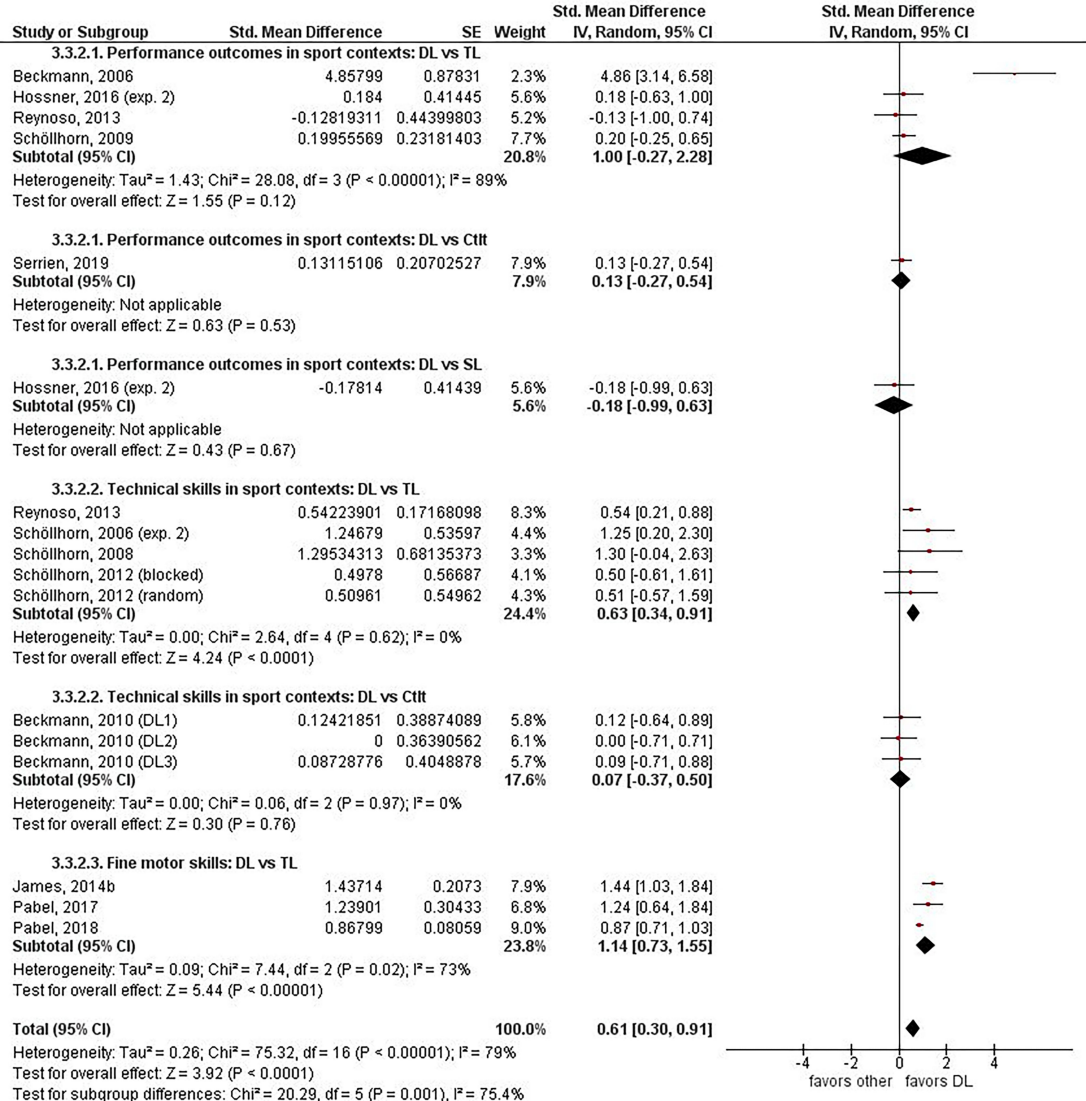
Learning phase (retention – pre). Forest plot for the effects of differential learning vs. other methods grouped by category of movement task. DL, differential learning; TL, traditional learning; CtIt, contextual interference; SL, structural learning.

To compare the effects of DL vs. other motor learning methods, effect sizes were extracted from the original research papers and grouped according to relevant context and outcomes. All data on individual effect sizes, 95% CI, overall estimated effect sizes, and heterogeneity are presented in Figure 2 (acquisition phase) and Figure 3 (learning phase). Given the relatively low number of experiments and heterogeneity between them, no further selection on quality was done and all experiments that provided data were used in the meta-analysis.

#### Acquisition Phase (Post – Pre, in Accordance With q30)

The forest plot of the acquisition phase can be found in Figure 2. Twenty-seven experiments reported the effects of DL in the acquisition phase compared to other motor learning methods (Schöllhorn et al., 2004, 2006, 2008, 2009b, 2010b; Beckmann and Schöllhorn, 2006; Beckmann et al., 2010; Savelsbergh et al., 2010; Reynoso et al., 2013; James, 2014; James and Conatser, 2014; Mateus et al., 2015; Hossner et al., 2016b; Pabel et al., 2017, 2018; Santos et al., 2017, 2018; Bozkurt, 2018; Coutinho et al., 2018; Gaspar et al., 2019; Ozuak and Çaglayan, 2019; Serrien et al., 2019). The overall effect was small and in favor of DL (*d* = 0.27, 95% CI = [0.12–0.42], *p* = 0.0006), and the test for overall subgroup differences was statistically significant (χ^2^ = 15.7, *p* = 0.02, *I*^2^ = 61.7%), indicating different effects of DL among the several subgroup analyses.

##### Performance Outcomes in Sport Contexts

Nine experiments were included in this subgroup analysis (Beckmann and Schöllhorn, 2006; Schöllhorn et al., 2009b, 2010b; Savelsbergh et al., 2010; Reynoso et al., 2013; Hossner et al., 2016b; Coutinho et al., 2018; Gaspar et al., 2019; Serrien et al., 2019). Participants in the DL group showed greater improvements from pre- to post-test than those in the TL group in seven of the eight experiments with a relatively small overall effect size (*d* = 0.37, 95% CI = [0.05–0.69], *I*^2^ = 58%). The study of Beckmann and Schöllhorn (2006) was considered an outlier across the entire meta-analysis. Only one study compared performance outcomes after SL to DL, with participants in the DL group showing less improvement than participants in the SL group (*d* = −0.19, 95% CI = [−1.00, 0.62]) (Hossner et al., 2016b). Also, one single study compared performance outcomes after CtIt to DL, with participants exposed to DL showing greater improvement than the CtIt group (*d* = 0.98, 95% CI = [0.56–1.40]) (Serrien et al., 2019).

##### Technical Skills in Sport Contexts

Fourteen experiments documented the effects of DL compared to TL (Schöllhorn et al., 2004, 2006, 2008, 2012; Reynoso et al., 2013; Mateus et al., 2015; Hossner et al., 2016b; Santos et al., 2017; Bozkurt, 2018; Coutinho et al., 2018; Gaspar et al., 2019; Ozuak and Çaglayan, 2019). Participants in the DL group showed on average greater improvements from pre- to post-test than participants exposed to TL in 12 out of the 14 experiments. The overall effect size and 95% CI was positive but small (*d* = 0.34, 95% CI = [0.17–0.51], *I*^2^ = 30%). Subgroup analysis on one study evaluating the effects of DL compared to CtIt revealed a negligible negative effect size (*d* = −0.04, 95% CI = [−0.48, 0.39]) (Beckmann et al., 2010).

##### Tactical Behavior in Sport Contexts

Four experiments were included in this subgroup analysis, showing a small positive overall effect size (*d* = 0.20, 95% CI = [−0.03, 0.44], *I*^2^ = 77%) with the DL group showing on average greater improvements from pre- to post-test in two of the four experiments (Mateus et al., 2015; Santos et al., 2017, 2018; Coutinho et al., 2018).

##### Fine Motor Skills

This subgroup analysis encompassed four experiments evaluating the effects of DL compared to TL (James, 2014; James and Conatser, 2014; Pabel et al., 2017, 2018). On average, participants in the DL group showed greater improvements from pre- to post-test than those in the TL group in three of the four experiments, but the overall effect size was negative but negligible (*d* = −0.12, 95% CI = [−1.04, 0.79]; *I*^2^ = 97%).

#### Learning Phase (Retention – Pre, in Accordance With q30)

The forest plot of the acquisition phase can be found in Figure 3. Twelve experiments reported the effects of DL in the retention phase compared to other motor learning methods (Beckmann and Schöllhorn, 2006; Schöllhorn et al., 2006, 2008, 2009b, 2012; Beckmann et al., 2010; Reynoso et al., 2013; James and Conatser, 2014; Hossner et al., 2016b; Pabel et al., 2017, 2018; Serrien et al., 2019). Not one experiment or outcome encompassed tactical behavior. The overall effect size was moderate in strength and in favor of DL (*d* = 0.61, 95% CI = [0.30–0.91], *p* < 0.0001) and the test for overall subgroup differences was statistically significant at the 5% level (χ^2^ = 20.29, *p* = 0.001, *I*^2^ = 75%) indicating different effects of DL among the several subgroup analyses.

##### Performance Outcomes in Sport Contexts

Six experiments were included in total, with four of them looking into DL-TL comparisons, only one experiment examining DL-CtIt, and one other researching DL-SL (Beckmann and Schöllhorn, 2006; Schöllhorn et al., 2009b; Reynoso et al., 2013; Hossner et al., 2016b; Serrien et al., 2019). Participants in the DL group demonstrated on average greater improvements from pre- to retention test than participants in the TL group in three of the four experiments with an overall large positive effect size (*d* = 1.00, 95% CI = [−0.27, 2.28], *I*^2^ = 89%) (Beckmann and Schöllhorn, 2006; Schöllhorn et al., 2009b; Reynoso et al., 2013; Hossner et al., 2016b). Only one study compared performance outcomes of DL to SL, with participants in the DL group showing on average less improvement with a negligible negative effect size (*d* = −0.18, 95% CI = [−0.99, 0.63]) (Hossner et al., 2016b). Also, one study compared performance outcomes after CtIt to DL, with the DL group showing negligible more improvement from pre- to retention test compared to CtIt (*d* = 0.13, 95% CI = [−0.27, 0.54]) (Serrien et al., 2019).

##### Technical Skills in Sport Contexts

Subgroup analysis on four experiments evaluating the effects of DL compared to TL showed on average stronger improvements from pre- to retention tests for the DL group (*d* = 0.63, 95% CI = [0.34–0.91]) (Schöllhorn et al., 2006, 2008, 2012; Reynoso et al., 2013). When comparing DL to CtIt for technical skills, only one study could be included, and a negligible effect of DL compared to CtIt was observed (*d* = 0.07, 95% CI = [−0.37, 0.50], *I*^2^ = 0%) (Beckmann et al., 2010).

##### Fine Motor Skills

Three experiments were included in this subgroup analysis and all studies showed superior improvements from pre- to retention test for DL compared to TL with large effect sizes (overall effect: *d* = 1.14, 95% CI = [0.73–1.55]) (James and Conatser, 2014; Pabel et al., 2017, 2018).

### Sensitivity Analyses

Table 3 presents the results of the sensitivity analyses on the calculation of the effect size variances, using various levels of the pre–post correlation. The results are fairly robust under a wide range of plausible correlation coefficients.

**Table 3:** Sensitivity analysis of the effect sizes [95% CI] based on various levels of the pre–post correlation coefficient.

**Acquisition phase**	**Pre–post correlation**		
	***r* = 0.15**	***r* = 0.50**	***r* = 0.85**
Performance outcomes in sport contexts: DL vs. TL	0.37 [0.03, 0.72]	0.37 [0.05, 0.69]	0.33 [0.08, 0.57]
Performance outcomes in sport contexts: DL vs. CtIt	0.98 [0.56, 1.40]	0.98 [0.56, 1.40]	0.98 [0.56, 1.40]
Performance outcomes in sport contexts: DL vs. SL	−0.19 [−1.25, 0.87]	−0.19 [−1.00, 0.62]	−0.19 [−0.64, 0.26]
Technical skills in sport contexts: DL vs. TL	0.35 [0.19, 0.52]	0.34 [0.17, 0.51]	0.35 [0.19, 0.51]
Technical skills in sport contexts: DL vs. CtIt	−0.04 [−0.61, 0.53]	−0.04 [−0.48, 0.39]	−0.04 [−0.28, 0.20]
Tactical behavior in sport contexts: DL vs. TL	0.17 [−0.07, 0.42]	0.20 [−0.03, 0.44]	0.14 [−0.24, 0.52]
Fine motor skills: DL vs. TL	−0.11 [−0.97, 0.74]	−0.12 [−1.04, 0.79]	−0.13 [−1.17, 0.90]
**Learning phase**	**Pre-retention correlation**		
	***r*** **= 0.15**	***r*** **= 0.50**	***r*** **= 0.85**
Performance outcomes in sport contexts: DL vs. TL	1.06 [−0.42, 2.53]	1.00 [−0.27, 2.28]	0.78 [−0.05, 1.61]
Performance outcomes in sport contexts: DL vs. CtIt	0.13 [−0.27, 0.54]	0.13 [−0.27, 0.54]	0.13 [−0.27, 0.54]
Performance outcomes in sport contexts: DL vs. SL	−0.18 [−1.24, 0.88]	−0.18 [−0.99, 0.63]	−0.18 [−0.63, 0.27]
Technical skills in sport contexts: DL vs. TL	0.65 [0.25, 1.04]	0.63 [0.34, 0.91]	0.69 [0.38, 1.00]
Technical skills in sport contexts: DL vs. CtIt	0.07 [−0.50, 0.63]	0.07 [−0.37, 0.50]	0.07 [−0.17, 0.31]
Fine motor skills: DL vs. TL	1.13 [0.73, 1.54]	1.14 [0.73, 1.55]	1.16 [0.73, 1.60]

*The default estimate of r = 0.50 is shown as reference (same as in forest plots and manuscript)*.

*DL, differential learning; TL, traditional learning; CtIt, contextual interference; SL, structural learning*.

### Publication Bias

**Figure 4:**
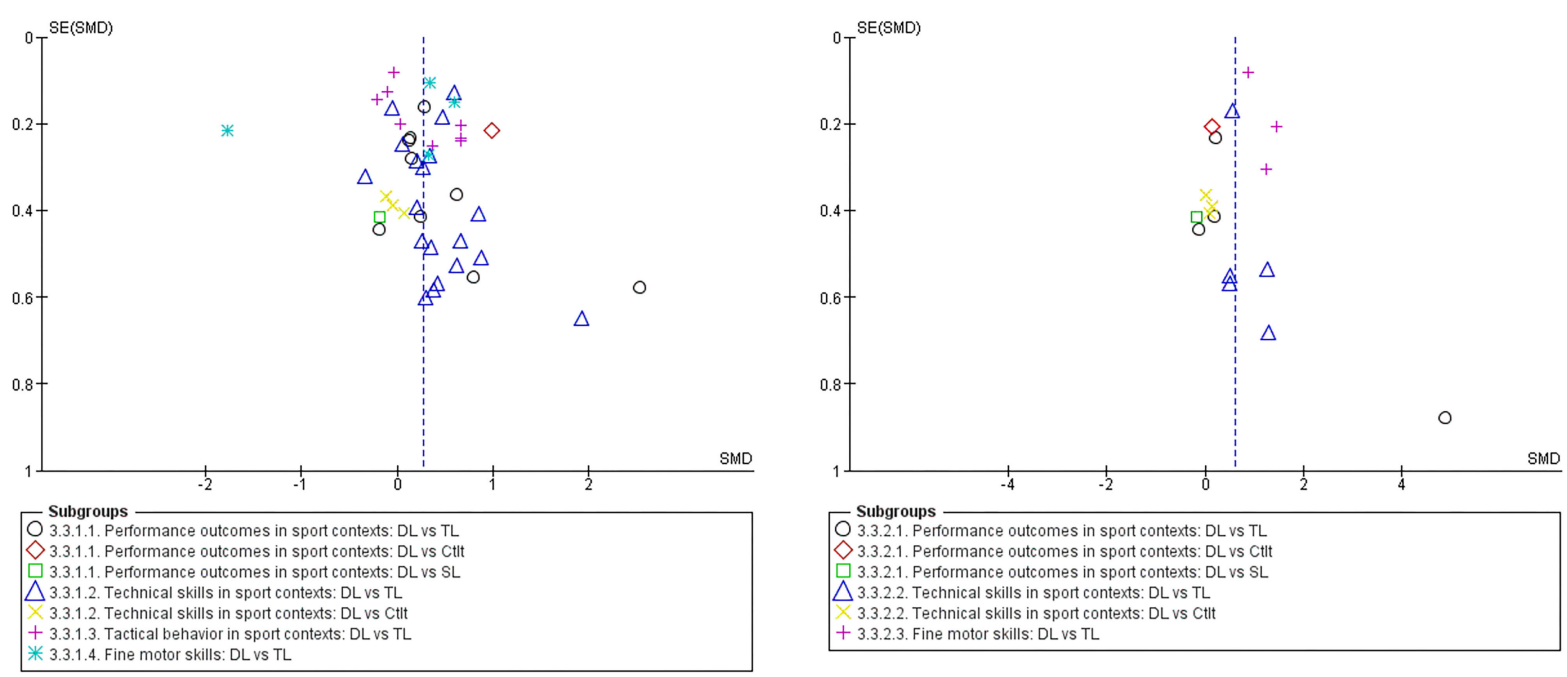
Funnel plots of the effect sizes of the acquisition phase (left) and learning phase (right). Vertical dashed line shows the overall effect size. DL, differential learning; TL, traditional learning; CtIt, contextual interference; SL, structural learning.

Figure 4 presents the funnel plot of all included studies. Visually, a moderate asymmetry toward the right is present in both funnel plots, but this is primarily due to the presence of strong outliers in both directions (Beckmann and Schöllhorn, 2006; Schöllhorn et al., 2006; James and Conatser, 2014). However, not every study could be included in the meta-analysis, which biases the interpretation of the funnel plots. In addition, the presence of many unpublished abstracts (e.g., https://sport.uni-mainz.de/publikationsliste/) indicates that publication bias is present and affected the results of the meta-analysis.

## Discussion

The objective of this meta-analytical review was to examine the evidence of studies that compared the effectiveness of DL to other motor learning methods in the performance of skills and movement tasks. We included 27 articles reporting outcomes of 31 experiments, with only 12 experiments documenting outcome measures in the retention phase. In the acquisition phase, DL is more effective compared to other motor learning methods with an overall small effect size of 0.27 [0.12, 0.42]. In the retention phase, however, DL appears on average to be more effective than other motor learning methods with an overall effect size of 0.61 [0.30, 0.91]. At first sight, one might be tempted to conclude that variability-based motor learning, DL in this case, culminates in higher improvements following practice than other motor learning methods (Frank et al., 2008; Lage et al., 2015; Schöllhorn and Horst, 2020). Nevertheless, it is important to emphasize that overall heterogeneity for the acquisition phase as well as for the retention phase was large, *I*^2^ = 78% and *I*^2^ = 79%, respectively. Also, the included papers in general had low sample sizes and showed high risk of bias and possible publication bias. The funnel plot (Figure 4) indicates that overall effect sizes should be carefully interpreted and warrants more high-quality research.

### Critical Interpretation on the Effects of DL in the Acquisition Phase

Bearing in mind that overall large heterogeneity (*p* < 0.00001, *I*^2^ = 78%) was found across the included studies, interpreting the results regarding improvements following practice of DL compared to other motor learning methods in the acquisition phase should be made with considerable care. At the subgroup level, concerning performance outcomes in sport contexts, DL showed higher improvements following practice than TL with a relatively small overall effect size. However, it is more than likely that the true effect size is lower, since the study of Beckmann and Schöllhorn (2006) had a strong influence on this subgroup's effect size. Heterogeneity between effects was large (*I*^2^ = 58%), indicating the presence of unexplained factors, such as the type of performance outcome (e.g., ice skating speed vs. throwing distance). Furthermore, the included studies did not show unanimous positive results, while the CIs for all studies, except the study of Beckmann and Schöllhorn (2006), crossed the line of null effect. Remarkably, the study of Hossner et al. (2016b, exp. 2) used a similar sample (size), similar context, duration, frequency, amount of exercises, and task as the study of Beckmann and Schöllhorn (2006) but the effect size was 10.5 times larger in the latter study than the former. Differences in the application of feedback and demonstrations probably contributed to these vastly different outcomes, although this alone might not sufficiently explain the big difference in effect sizes between these two studies. Moving on to another subgroup, DL might enable slightly higher improvements following practice in tactical behavior in sports. Nevertheless, also in this case large heterogeneity was present across the included experiments of this subgroup (*I*^2^ = 77%). This can be partially explained by differences in population (e.g., experience level, age) and used outcome measures (e.g., basketball vs. soccer). Another possible factor contributing to this high level of heterogeneity could have been the subjective nature and interpretation of some tactical variables (e.g., creative components). Although these studies were the first to research tactical outcome measures and play an important role in the development of motor learning research by providing insights in this previously unexplored area, more objective tactical outcome measures should be included in future research. Regarding fine motor skills, DL performed on average better than TL. Yet, the overall effect size was negative and the CI covered zero (*d* = −0.12, [−1.04, 0.79]) largely due to a strong negative outlier causing large heterogeneity (*I*^2^ = 97%). The “technical sport skills (DL vs. TL)” was the only subgroup with a relative low amount of heterogeneity (*I*^2^ = 30%). Here, a small positive effect was found for DL compared to TL. These results should nonetheless be interpreted with caution, as not all included studies demonstrated effects favoring the DL method; the CIs of the majority of studies crossed the line of null effect, and most of the experiments were carried out by the same research group. The results of three subgroups [DL vs. CtIt (sport performance outcomes), DL vs. CtIt (sport technical skill), and DL vs. SL (sport performance outcomes)] should not be interpreted separately, since an insufficient number of experiments (1) and participants were included in each subgroup.

In summary, the test for overall effect shows a statistically significant difference favoring DL over other motor learning methods in the acquisition phase (*p* = 0.0006). Nevertheless, as already stated above, to interpret this total summary, statistical results would be premature in light of the considerable amount of heterogeneity. Given that this information is less meaningful, it is recommended to devote more attention to the subgroup analyses. Three out of seven subgroups had very large variances due to low sample sizes, while three other subgroups only encompassed one study, which limits generalizability of the results. Therefore, the validity of the improvements following practice estimate for each subgroup is uncertain, as individual trial results are inconsistent. Despite the circumstantial and low-quality evidence, it seems that the acquisition could be slightly enhanced when applying DL in comparison to TL. When comparing DL to other variability-based motor learning methods (i.e., SL and CtIt), not one motor learning method currently appears to be superior for acquisition. Although it might be too early to assert these general statements, the discrepancy in results and the large heterogeneity proclaim the need for further high-quality research on this topic by independent research groups and clear demarcation of both the DL method and other motor learning methods.

### Critical Interpretation on the Effects of DL in the Retention Phase

Given that the overall heterogeneity was large across the included studies in the retention phase (*p* < 0.00001, *I*^2^ = 79%) and the amount of included experiments was low (*n* = 12), interpreting the results regarding improvements following practice of DL compared to other motor learning methods in the retention phase should be made with great caution if they are to be made at all. Comparable to the acquisition phase, similar disconcerting patterns emerge regarding heterogeneity, low sample sizes, low power, etc. Even though fewer studies could be included during the retention phase, averaged across all subgroup comparisons, the effect of DL was two to three times larger in the retention phase (*d* = 0.61, [0.30, 0.91]) compared to the acquisition phase (*d* = 0.26 [0.10, 0.42]). Nevertheless, readers should critically interpret and reflect on these effect sizes. Similar to the acquisition effect for shot put training, both studies of Beckmann and Schöllhorn (2006) and Hossner et al. (2016b) found a better learning effect for DL compared to TL, but a very large discrepancy was observed for the effect sizes. Despite their similar designs, the study of Beckmann and Schöllhorn (2006) demonstrated a 27 times larger effect size than the study of Hossner et al. (2016b). Mainly fine motor skills and sports technical skills seem to be better retained after DL intervention in comparison to TL. Although sensible interpretations should be made on these two topics. The sport technical skills subgroup mainly encompassed studies from one research group with the CIs of some studies exceeding the line of null effect, while the fine motor skills subgroup encompassed a large amount of heterogeneity (*I*^2^ = 73%). Furthermore, three out of seven subgroups (all DL vs. other variability-based motor learning methods) could only include one study, which implicates very low generalizability and minimal attributable value to potential inferences based on these results. Nevertheless, the result of the overall effect shows a statistically significant difference favoring DL over other motor learning methods (*p* < 0.0001). However, general interpretations about the effectiveness of DL compared to other motor learning methods in the retention phase should be made with great caution. This is due to the large amount of heterogeneity, the limited number of studies, low sample sizes, and considerable risk of bias across all studies.

### Does the Current Empirical Evidence on DL Support Its Theoretical Rationale and the Variability-Based Continuum?

The findings of the meta-analysis are partly in line with the theoretical rationale of DL that strives to achieve an individual optimal level of variability in practice, allowing the athlete to discover different aspects of his/her dynamic movement landscape and withhold the most efficient and effective movement solution as part of the motor learning process. Recently, the DL method received a high degree of attention in research and practice, partly due to its hypothesis of potentially being an enhanced motor learning method (= provides the learner with a higher learning rate than other methods), partly due to researchers' critical attitude toward the DL method (Pabel et al., 2017, 2018; Bozkurt, 2018; Coutinho et al., 2018; Santos et al., 2018; Gokeler et al., 2019; Serrien et al., 2019; Weisner et al., 2019).

The differences of DL with other methods that employ practice variability are the amount and/or structure of the exercise variations. Schöllhorn et al. (2009a) depicted various motor learning methods in a continuum of increasing variability and noise (REP, MSE, VP, CtIt, CLA, SL, and DL) with DL being hypothesized to exemplify the highest learning rates (Schöllhorn et al., 2009a; Schöllhorn and Horst, 2020). However, the results of the current meta-analysis question the validity of this continuum. For a robust comparison of DL to other motor learning methods inspired by variability (VP, CtIt, CLA, SL), scarce and inconclusive evidence exists to examine and infer whether DL is superior or inferior in terms of learning rate. Additionally, we want to draw attention to the difficulty in distinguishing between DL and SL (Hossner et al., 2016b; Schöllhorn, 2016). Both methods use a large overall practice variability, but SL tries to minimize trial-to-trial variability (subsequent exercises are different in only a small detail). This led to the terminology of “gradual DL” as synonym for SL and “chaotic DL” for the classical interpretation that uses random trial-to-trial variability (Henz et al., 2018; Schöllhorn and Horst, 2020).

Based on the meta-analyses and in light of the low methodological quality of the included studies, DL shows potential to be considered as an enhanced motor learning method in comparison to TL methods when aiming to improve motor learning during the acquisition and retention phase. For both the acquisition and retention effect, the study with the lowest risk of bias (Pabel et al., 2018) was in line with the subgroup and omnibus effect size estimate.

Furthermore, the theory and mechanism behind the DL method is not undebated (Schoner, 1995; Scholz and Schöner, 1999; Latash et al., 2007; Beek, 2011; Künzell and Hossner, 2012, 2013; Schmidt and Hennig, 2012; Willimczik, 2013; Schöllhorn et al., 2015; Hossner et al., 2016a; Schöllhorn, 2016). Nevertheless, a detailed discussion on the theoretical background, key features, underlying (supposed) mechanisms, predictions, and limitations of DL in comparison to other motor learning methods is beyond the exploratory and practical focus of this systematic review and meta-analysis. Readers should thus also be aware of the following key points when interpreting the results of this study: (1) some fundamental limitations exist with the theoretical framework of DL, (2) DL studies are mostly focused on learning effectiveness rather than learning rate and that the effectiveness is assessed imperfectly when a pre- to post-test design is used rather than a design that also includes a retention/transfer test, (3) there are alternative methods available that predict benefits of VP but for different reasons than DL (e.g., schema theory, uncontrolled manifold hypothesis), and (4) CtIt and SL can be used to schedule VP.

### How Can These Results Impact Motor Learning in Sport or Rehabilitation Contexts?

Trainers and clinicians often merge different theoretical motor learning concepts with the aim to improve athletes' or patients' motor or movement skill performance. The results of this meta-analysis do not allow for strong recommendations in favor of a specific motor learning method toward trainers or clinicians. However, a well-considered use of (increasing) variability appears to be beneficial over more traditional or repetitive motor learning methods. Farrow and Robertson (2017) discuss the role of variability-based learning within a skill acquisition periodization framework. They stress the role of variability in countering tedium, but refrain from giving general guidelines on where in the periodization of micro-, meso-, and macrocycles this is most optimal as the literature is not able to substantiate evidence-based criteria. In line with the model of Schöllhorn and Horst (2020), Farrow and Robertson (2017) propose a practical continuum of variability that can be offered to athletes, trainers, clinicians, and researchers.

Important in real-world training situations, whether it be performance or clinically oriented, is to shift focus toward individuality and specificity. Other important variables such as instruction, feedback, focus of attention, motivation, etc should also be considered besides the amount and structure of provided variability since these variables have also been shown to play an important role in motor learning in sport and rehabilitation contexts (Wulf and Lewthwaite, 2016; Gokeler et al., 2019). In a sport context, the integration of variability in motor learning possibly promotes motivation by increasing the challenge of training (Guadagnoli and Lee, 2004) as well as promoting fun and enhanced expectancies during practice (Wulf and Lewthwaite, 2016). In a clinical context, focusing on the current capacity, the individual needs and goals of the patient are essential in order to select the most fitting motor learning method. Implementing insights from DL (together with other variability-based motor learning methods) and a well-considered use of variability can improve task performance on the short term allowing for enhanced motor learning during the acquisition phase, while fine motor skills likely benefit the most from the retention effect of DL (Pabel et al., 2017, 2018). Restoring gross and fine motor skills are an important aspect of neurological and musculoskeletal rehabilitation given the known persistence of sensorimotor impairments (Repšaite et al., 2015; Gokeler et al., 2019). Increasing variability in rehabilitation should always be performed in a safe context, allowing for successful but challenging exercises to allow the patient to explore efficient and effective movement strategies that transfer to real-world scenarios (Guadagnoli and Lee, 2004). Nevertheless, data on the application of DL during a rehabilitation process after injury or in a sport injury risk mitigation plan is scarce to non-existent.

In training/rehabilitation contexts, the learning of a single movement is rarely the goal. Regarding transfer effects, many experiments that were included assessed the effects of DL on more than one movement (Schöllhorn et al., 2012) or included several different outcome variables of the same movement (Reynoso et al., 2013) or outcome variables from different movements (Mateus et al., 2015; Santos et al., 2017, 2018). Studies that explicitly used a transfer test (e.g., Beckmann et al., 2010) were scarce and not included in any meta-analysis. DL uses variability with the aim to prepare subjects to be able to cope with a large range of unforeseen situations (Schöllhorn et al., 2010a); therefore, we recommend future studies to address transfer effects to unforeseen situations or to related movements.

### Limitations

Publication bias and missing data for the meta-analysis may have influenced the results. The meta-analysis was based on a very heterogeneous sample of studies with widely varying populations, motor tasks, and control conditions. These high levels of heterogeneity stress the importance to interpret these results with caution and call for high-quality future research. For the acquisition phase, the subgroups based on type of task and type of control condition were a significant factor in explaining the heterogeneity. However, only one study compared DL to SL (Hossner et al., 2016b), while one study compared it to CtIt, and all others compared it to TL. Future analyses may consider further subgroups for REP and MSE comparisons. Regarding heterogeneity in sample characteristics, future analyses must consider additional subgroup analyses based on age and/or level of expertise as we grouped results from complete novices and experts in the same analysis. Also, dividing the meta-analysis into different subgroups based on the type of task (e.g., performance, technical skill) might not be ideal for a holistic interpretation on this topic, though an overall effect size was calculated for both the overall acquisition and retention phase. From a theoretical perspective, the most important covariate to be considered in future meta-analyses is likely the noise level of the training intervention. A difficulty here will be to find a proper common metric that quantifies this outcome.

Besides co-interventions representing general practice in non-laboratory contexts and being in line with representative learning design directives to ensure functionality and action fidelity in training and learning environments (Pinder et al., 2011), the inclusion of experiments with co-interventions (Mateus et al., 2015; Repšaite et al., 2015; Santos et al., 2017) might also be a potential confounder of the results. However, as noted earlier, in practical contexts, several methods are often combined, so these experiments can provide important information. Furthermore, studies without assessment of performance variables (Menayo et al., 2014; Henz and Schöllhorn, 2016; Henz et al., 2018) were not included in this meta-analysis although they provide valuable information on specific aspects of DL. These studies are especially important for inquiry about the individuality and situation specificity of the stochastic resonance.

A final limitation is the unknown pre–post and pre-retention correlations in the study reports. The sensitivity analysis showed that this parameter had only a small influence on the overall effect sizes and their 95% CI, but this assumed a fixed correlation coefficient across all studies and may potentially have a larger influence. The overview of effect sizes and their 95% CI may be used in the design of future interventions.

### Implications for Future Research

In general, further high-quality research is necessary with low risk of bias RCTs and publications in peer-reviewed journals (Beek, 2011). Given the nature of motor learning experiments, it is challenging and, in many cases, impossible to blind participants, researchers, trainers, and therapists to which condition they are assigned to. Therefore, future studies should make a bigger effort in addressing the other risk of bias items in their study design and report them accordingly. Also a major recommendation for future research is to better define, design, and report the used control conditions in the study of DL. When motor learning refers to the study of cognitive, perceptual, motor, and physiological responses that explain motor skill acquisition, more attention should be devoted to the retention effects of motor learning interventions both in the short term and in the long term. Future research should also aim to encompass more robust designs, increase sample sizes, and clearly define the motor learning method that is experimentally tested as well as the motor learning method used to compare with, and to be published in international peer-reviewed journals. In particular, studies researching the differences between variability-based methods (DL, SL, CtIt, VP, and CLA) at the theoretical and the practical level are much needed. Potential interesting variables to address in future research could be the amount and structure of applied variability. Besides variability, other variables like instruction, feedback, focus of attention, motivation, level of expertise, etc should also be considered. Given the focus on individuality in DL, it will be important to study the relationships between dose (variability) and response (learning rate), and to identify factors that predict optimal amounts in specific populations and situations (Caballero et al., 2017). Also, the problem on the role of variability in motor learning periodization requires further investigations (Farrow and Robertson, 2017). Single-subject analyses may prove valuable for these fundamental questions.

## Conclusion

Given the large amount of heterogeneity, low availability of studies, low sample sizes, and considerable risk of bias across all studies, inferences about the effectiveness of DL should be made with prudence. Considering these methodological flaws, DL shows potential to be considered as an enhanced motor learning method in comparison to TL methods when aiming to improve motor learning in the acquisition and retention phase. A robust comparison and conclusion on the relative effectiveness of DL to other motor learning methods inspired by variability (i.e., SL and CtIt) would be premature, since scarce and inconclusive evidence was found. Future research should aim to perform more high-quality research. Once more high-quality research becomes available, the results of this meta-analysis should be updated in combination with stricter inclusion criteria concerning study design, risk of bias, and publication policy.

## Data Availability Statement

Publicly available datasets were analyzed in this study. This data can be found here: https://osf.io/m4sje/.

## Author Contributions

The conception and drafting of the work and the acquisition and the analysis of data were carried out by BT and BS. All authors made substantial contributions to the design of and interpretation of data for the work. All authors revised the manuscript critically for important intellectual content and provided approval for publication of the content. All authors agreed to be accountable for all aspects of the work in ensuring that questions related to the accuracy or integrity of any part of the work are appropriately investigated and resolved.

## Conflict of Interest

The authors declare that the research was conducted in the absence of any commercial or financial relationships that could be construed as a potential conflict of interest.

## Footnotes

^1^https://www.youtube.com/watch?v=U2AMfyyUt5c.
